# Urine metabolites for the identification of *Onchocerca volvulus* infections in patients from Cameroon

**DOI:** 10.1186/s13071-021-04893-1

**Published:** 2021-08-11

**Authors:** Vera Wewer, Helga Peisker, Katharina Gutbrod, Mazen Al-Bahra, Dirk Menche, Ngongeh Glory Amambo, Fanny F. Fombad, Abdel Jelil Njouendou, Kenneth Pfarr, Samuel Wanji, Achim Hoerauf, Peter Dörmann

**Affiliations:** 1 Institute of Medical Microbiology, Immunology and Parasitology (IMMIP), University Hospital Bonn, Venusberg-Campus 1, 53127 Bonn, Germany; 2 Institute of Molecular Physiology and Biotechnology of Plants, University of Bonn, Karlrobert-Kreiten-Str. 13, 53115 Bonn, Germany; 3 Kekulé-Institute of Organic Chemistry and Biochemistry, University of Bonn, Gerhard-Domagk-Str. 1, 53121 Bonn, Germany; 4 Present Address: Center of Excellence in Plant Sciences (CEPLAS), Mass Spectrometry Platform, University of Cologne, Zülpicher Str. 47b, 50674 Cologne, Germany; 5 German Center for Infection Research (DZIF), Partner Site Bonn-Cologne, Bonn, Germany; 6 Research Foundation for Tropical Diseases and the Environment (REFOTDE), Buea, Cameroon; 7 Parasite and Vector Research Unit (PAVRU), Department of Microbiology and Parasitology, University of Buea, Buea, Cameroon

**Keywords:** *Onchocerca volvulus*, NATOG, Mass spectrometry, Diagnosis, Filariasis, Onchocerciasis, Metabolite

## Abstract

**Background:**

The tropical disease onchocerciasis (river blindness), caused by *Onchocerca volvulus* filarial nematodes, is targeted for elimination by mass treatment with nematocidal and antimicrobial drugs. Diagnosis of *O. volvulus* infections is based on counts of skin-borne microfilariae, but additional diagnostic tools, e.g. worm- or host-derived small RNAs, proteins or metabolites, are required for high-throughput screening. *N*-acetyltyramine-*O*,β-glucuronide (NATOG) was suggested as a biomarker for onchocerciasis but its viability as diagnostic tool has been challenged.

**Methods:**

We performed a screening program of urine samples from individuals from Cameroon infected with *O. volvulus*, *Loa loa*, *Mansonella perstans* or a combination thereof. Urine metabolites were measured by liquid chromatography–mass spectrometry (LC-MS). Principle component analysis (PCA) revealed that onchocerciasis causes complex changes of the urine metabolome.

**Results:**

The mean NATOG content was elevated in urine of *O. volvulus*-infected compared with non-infected individuals, but NATOG levels showed considerable variation. However, 13.8% of all *O. volvulus*-infected individuals had high NATOG levels never reached by individuals without filarial infections or only infected with *L. loa* or *M. perstans*. Therefore, the identification of individuals with high NATOG levels might be used to screen for the elimination of onchocerciasis after mass drug application. Additional metabolites, including a compound identified as cinnamoylglycine, had high PC1/PC2 loadings in the data set. Mean levels of cinnamoylglycine were increased in *O. volvulus*-infected individuals, and 17.2% of all *O. volvulus* individuals had elevated cinnamoylglycine levels not reached by the controls.

**Conclusions:**

On an individual level, NATOG alone had poor discriminative power distinguishing infected from non-infected individuals. However, 13.8% of all *O. volvulus*-infected individuals had NATOG levels never reached by individuals without filarial infections or infected with only *L. loa* or *M. perstans*. Discrimination of *O. volvulus* infections from controls or individuals suffering from multiple infections was improved by the measurement of additional metabolites, e.g. cinnamoylglycine. Thus, measuring a combination of urine metabolites may provide a way to assess onchocerciasis on the population level. This provides the possibility to design a strategy for large-scale onchocerciasis epidemiological screening programs based on urine rather than invasive techniques.

**Graphical abstract:**

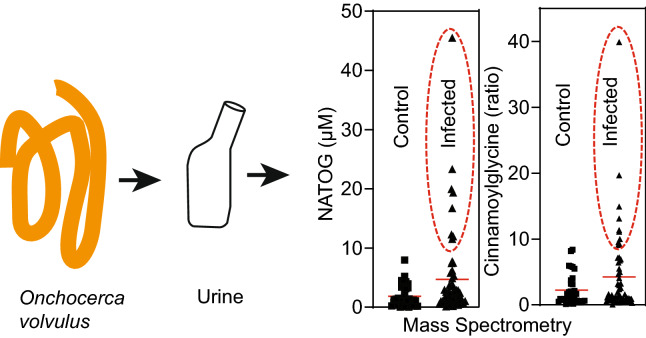

**Supplementary Information:**

The online version contains supplementary material available at 10.1186/s13071-021-04893-1.

## Background

Filarial infections transmitted via insect vectors are still prevalent in many parts of West and Central Africa. Among these, *Onchocerca volvulus*, which causes onchocerciasis (river blindness) and is transmitted through bites of the black fly, infects patients mostly in rural areas of West Africa [1]. The juvenile worms migrate to subcutaneous tissue and form nodules. The adult males mate with the females in the subcutaneous tissue, and the female produces microfilaria that migrate through the subcutaneous tissue. In addition to *O. volvulus*, further filarial infections are endemic in the affected areas, e.g. *Loa loa* and *Mansonella perstans* [2]. Filarial infections, in particular onchocerciasis, are part of the list of 20 neglected tropical diseases targeted for elimination by the WHO by 2030 [3]. Nodule palpation and skin snips with microscopic observation of microfilariae are still the gold standard for onchocerciasis. Real-time PCR is also available, but is mostly used on identifying *O. volvulus* nematodes in the black fly vectors. As these tools are laborious in large-scale screening strategies following mass drug administration programs, efforts to identify alternative markers for onchocerciasis and related infections were initiated [4].

Several alternatives to skin snips/palpation depend on the identification of antibodies recognizing *O. volvulus* proteins. These serological methods allow for rapid screening with non-invasive sample collection. However, with successful mass drug treatment, the assays lose power as fewer positive cases are identified, leading to a large spread in confidence intervals, resulting in a borderline definition. Thus, second-stage mapping with larger sample sizes is required to determine whether the borderline cases indicate continued prevalence of *O. volvulus* infections. This also increases the logistical costs and delays decision making. Rather than second-stage mapping, screening for other biomarkers could be included to break the borderline classification.

The presence of microfilaria or juvenile/adult worms in subcutaneous tissue is expected to result in the accumulation of filarial molecules or of compounds produced in the host in response to filarial infection. In line with this scenario, different worm-derived RNAs and proteins/peptides were detected in the bloodstream [5–7]. While such compounds are detectable in the host after infection, their concentration in the blood might be too low to be used as a diagnostic tool [8]. Small molecules represent an alternative tool for the diagnosis of onchocerciasis in the blood and in urine samples [9–11]. Denery et al. [9] were the first to introduce the concept of metabolite biomarkers to the diagnosis of onchocerciasis. Fourteen metabolites were identified in human plasma and serum by liquid chromatography-mass spectrometry (LC-MS) as potential *Onchocerca* biomarkers. Based on these studies, a search for biomarkers in human urine samples resulted in the identification of *N*-acetyltyramine-*O*,β-glucuronide (NATOG) as the most promising candidate [10]. The mean NATOG content was elevated in urine samples of onchocerciasis-positive patients from Africa compared with the mean of control samples. NATOG represents the glucuronidated and acetylated form of tyramine, a neurotransmitter of *O. volvulus*. Therefore, it was hypothesized that NATOG accumulation in the plasma might be caused by the derivatization of a worm-derived molecule in the host. Later, NATOG was also identified as a biomarker during the infection of jirds (*Meriones unguiculatus*) with *Litomosoides sigmodontis*, a related filarial nematode of rodents [12].

After screening additional human urine samples via LC-MS, NATOG was suggested to serve as a valid biomarker, and a threshold of 13 µM NATOG was proposed to discriminate between infected and non-infected individuals [13]. An improved NATOG assay based on a lateral flow immunoassay was introduced because the LC-MS method is difficult to apply in the field [14]. The usefulness of NATOG as a biomarker for onchocerciasis was later challenged because NATOG could not discriminate between urine samples from infected and non-infected individuals from a low endemic setting [15]. Recently, Hotterbeekx et al. [16] came to a similar conclusion. Only when subsets of patients with specific symptoms (i.e. severe form of epilepsy) were studied was the mean of NATOG levels considerably increased compared with non-infected controls.

To evaluate the viability of NATOG or other metabolite biomarkers, we measured NATOG in urine samples from patients from Cameroon infected with *O. volvulus*, *L. loa*, *M. perstans* or a combination of the three filarial diseases. The threshold of 10 µM NATOG was never surpassed by individuals infected only with *L. loa* or *M. perstans*. However, in 13.8% of those infected with *O. volvulus*, either as monoinfection or together with *M. perstans* or *L. loa* infections, NATOG was elevated above 10 µM. This would allow using NATOG as an epidemiological marker for geographical mapping of onchocerciasis. Subsets of metabolites were used for the analysis of their power to discriminate between infected and non-infected samples. One additional urine metabolite, cinnamoylglycine, also had elevated levels in infected individuals. Therefore, *O. volvulus* and other filarial diseases affect the urine metabolome pattern in a complex way, and the combination of more than one biomarker might help to differentiate between infected and non-infected individuals.

## Methods

### Urine samples

Urine samples from Cameroonian patients were collected and archived as part of the Bill & Melinda Gates Foundation consortium project "Rapid and high throughput diagnosis of *Onchocerca volvulus* infections" (RADIO; OPP1083888; Additional file 1: Table S1). Infected individuals were identified by palpation of onchocercomata and identification of microfilariae by microscopy of skin biopsies or blood as described [17–19]. The sample set included urine from uninfected participants; patients infected with *O. volvulus*, *L. loa* or *M. perstans*; or patients infected with two or more of the three filarial infections examined. Participants were provided sterile wipes and instructions on how best to collect urine free of skin contaminants. Collected urine was refrigerated within 5 min of collection for transport to the laboratory, where it was aliquoted under a laminar flow hood and frozen at − 80 °C.

### Metabolite extraction

After thawing, aliquots were taken from the urine samples for extraction with methanol. All samples were treated in the same way during analysis. Urine samples were centrifuged and 100 µl of the supernatant was mixed with 400 µl ice-cold methanol and internal standard (D_3_-NATOG, 20 µl in methanol; 0.36 nmol). D3-NATOG was synthesized as published [10] (Additional file 2: Text 1 and Additional file 3: Figure S1). Samples were vortexed and again centrifuged for 10 min at 13,000*g*. The supernatants were transferred to new microfuge tubes and dried in a SpeedVac for 1.5 h. Then, 300 µl of ice-cold methanol was added, and the samples were vortexed. Insoluble components were removed by centrifugation (10 min, 13,000*g*). The supernatants were transferred to autosampler vials (glass inlets) and the solvent removed in a SpeedVac. Samples were dissolved in 100 µl water/methanol (95:5).

### Liquid chromatography mass spectrometry

The samples were analyzed by LC-MS using an Agilent quadrupole time-of-flight (Q-TOF) instrument (Agilent 6530 AccurateMassQ-TOF) equipped with an electrospray ionization (ESI) source. The samples were separated on a Supelcosil column (ABZ+plus, 10 cm × 2.1 mm, 3 µm particle size) using a gradient elution as previously described [10]. Data were recorded and analyzed using the Agilent MassHunter (Qualitative Analysis) software. The peaks for NATOG, D_3_-NATOG and creatinine were identified after injecting pure standards according to their retention times and mass spectra. D_3_-NATOG was eluted at 19.5–20.5 min and quantified after fragmentation (transition of *m*/*z* 359.157 to 121.065). NATOG had the same retention time and was quantified using the transition of 356.134 to 121.065. Creatinine was measured without fragmentation during LC-MS with an elution time of 4.0–4.5 min with an *m*/*z* of 114.066.

### Data analysis and statistical methods

LC-MS data were uploaded to the XCMS online server at the Scripps Institute (https://xcmsonline.scripps.edu/news/xcmsserver.html) [20]. Principal component analysis (PCA) was done using the Statistical Analysis Tool of the RIKEN Center for Sustainable Resource Science (http://prime.psc.riken.jp/compms/others/main.html) (Kanagawa, Japan) [21]. Figures were constructed, and Mann-Whitney U test (with *P*-value exactly calculated) and Spearman rank test were performed with GraphPad Prism Version 9.1 (GraphPad Software, San Diego, CA, USA, www.graphpad.com) with significance set to *P* ≤ 0.05.

## Results

### NATOG as biomarker in urine samples of filariasis patients from Cameroon

To study the viability of employing small molecules, e.g. NATOG, as biomarkers for *O. volvulus* infection, metabolites were extracted from urine samples from infected (‘Ov-infected,’ *n* = 41) and non-infected (*n* = 24) individuals collected during a previous campaign in Cameroon (Additional file 1: Table S1). Further samples were included from individuals infected with other filarial infections (*Loa loa*, ‘Ll-infected,’ *n* = 6; *Mansonella perstans*, ‘Mp-infected,’ *n* = 5) and with multiple infections (*O. volvulus* and *L. loa*, ‘Ov + Ll-infected,’ *n* = 6; *O. volvulus* and *M. perstans*, ‘Ov + Mp-infected,’ *n* = 8; *O. volvulus*, *L. loa* and *M. perstans*, ‘Ov + Ll + Mp-infected,’ *n* = 3). The samples therefore included 58 individuals infected with *O. volvulus* alone or with an additional filarial nematode (‘all-Ov-infected’) and 35 samples from individuals without *O. volvulus* infection (non-infected and individuals carrying only *L. loa* or *M. perstans* infections; ‘all-controls’).

**Fig. 1:**
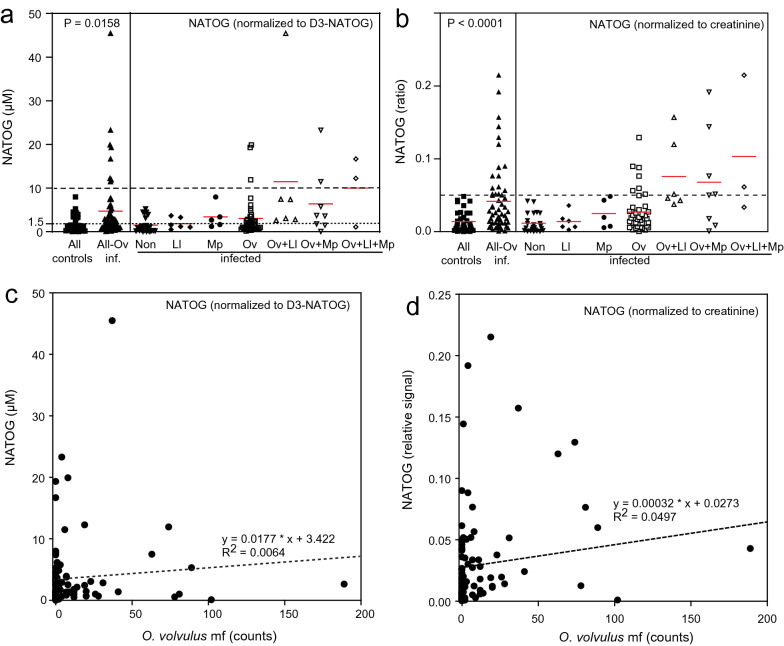
Amounts of NATOG in urine samples of individuals from Cameroon infected with filariasis. NATOG, D_3_-NATOG and creatinine were measured by LC-MS/MS. **a** NATOG (in µM) normalized to the internal standard D_3_-NATOG. The dashed and dotted lines depict the thresholds of 10 µM and 1.5 µM NATOG for diagnosis of *O. volvulus* infections, respectively. **b** NATOG normalized to creatinine (ratio of peak areas). The dashed line indicates a ratio of NATOG to creatinine of 0.05. The red lines indicate the means of the corresponding data. The data to the right of the vertical line contain results for non-infected, *Loa loa* (Ll)-infected, *Mansonella perstans* (Mp)-infected, *Onchocerca volvulus* (Ov)-infected, multiple infections (Ov, Ll, Mp). The data to the left of the vertical line contain the results for all controls (all non-infected samples and samples from individuals negative for Ov) and all *O. volvulus*-infected (Ov-, Ov + Ll, Ov + Mp and Ov + Ll + Mp-infected). Significance calculated with Mann-Whitney U test, GraphPad Prism version 9.1. **c** Correlation of NATOG contents (normalized to D3-NATOG) in urine with *O. volvulus* microfilarial load. The NATOG contents (in µM) in the urine samples were plotted vs. the number of *O. volvulus* microfilaria (Additional file 4: Table S1). **d** Correlation of NATOG contents normalized to creatinine in urine with *O. volvulus* microfilarial load. The NATOG contents (peak area ratios) in the urine samples were plotted vs. the number of *O. volvulus* microfilaria. The dashed lines show the regression lines and the *R*^2^ values. Note that samples with 0 microfilaria include all non-infected, Ll-infected, Mp-infected and some Ov-infected and multiple-infected samples

Metabolites were extracted in the presence of the internal standard D_3_-NATOG and quantified as previously described [10]. The NATOG measurements were performed with amounts in the linear range of quantification of 0.01 to 10 nmol in 100 µl sample (Additional file 4: Figure S2). The NATOG concentration in urine is shown in Fig. 1a. The all-Ov-infected samples were significantly different compared with all-controls (Mann-Whitney U test: *U* = 712, *P* = 0.0158). While the NATOG mean ± SEM concentration in Ov-infected (3.0 ± 0.7 µM) was increased compared with non-infected samples (1.5 ± 0.3 µM), a large variation was observed in the two groups. Using the threshold of 10 µM (dashed line in Fig. 1a), close to the previously proposed 13 µM [13]), all non-infected samples were below this threshold, while 3 of 41 Ov-infected samples and 5 of 17 of the co-infected individuals contained NATOG levels > 10 µM. Taken together, none of the non-infected, but 8 out of 58 all-Ov-infected (13.8%) had NATOG levels > 10 µM. When the concentration of 1.5 µM (mean of all non-infected samples) was used as a lower threshold, 20 out of 41 Ov-infected had higher NATOG levels, but only 6 out of 24 samples of the non-infected controls were above this threshold. Furthermore, the mean NATOG content in the Ov-infected, Ov + Ll-infected, Ov + Mp-infected and Ov + Ll + Mp-infected samples were also elevated compared with the non-infected samples. NATOG levels in the all-Ov-infected samples (Ov-infected, Ov + Ll-infected, Ov + Mp-infected, Ov + Ll + Mp-infected) varied from ~ 0 to > 45 µM, and 14 of 17 samples (82%) of the multiple infected individuals had NATOG levels > 1.5 µM.

The NATOG concentration showed a considerable variation and seemed to be partially independent of the infection status (Fig. 1a). We considered the possibility that different dilutions of urine compounds might have affected the NATOG measurements. Therefore, the NATOG peaks were normalized to creatinine (measured by LC-MS in the same run) to compensate for the fact that urine samples might contain different amounts of water (Fig. 1b) [15]. While individual data points were shifted, the general pattern of NATOG levels in infected and non-infected individuals remained similar. When normalized to creatinine, the all-Ov-infected samples were significantly higher compared with the all-controls (Mann-Whitney U test: *U* = 527, *P* < 0.0001). When a threshold of 0.05 was selected for the NATOG levels normalized to creatinine, 100% of the all-control samples were below this ratio, while 17/58 (29.3%) of the all-Ov-infected samples had NATOG levels ≥ 0.05 (Fig. 1b).

Because it was possible that the accumulation of NATOG in the urine sample might depend on the microfilarial load of *O. volvulus*, the NATOG levels were plotted against the microfilaria counts (Additional file 5: Table S2, Fig. 1c, d). For this analysis, all samples (including Ll-infected, Mp-infected and individuals with multiple infections) were included. However, the NATOG levels (in µM) did not correlate with the microfilarial load (Spearman *r* = 0.0846, *P* = 0.16). In addition, NATOG levels also did not correlate with the number of nodules (Additional file 1: Table S1). The correlation of NATOG normalized to the creatinine concentration in the urine with the microfilarial count was significant (Fig. 1d) (Spearman *r* = 0.3350, *P* = 0.001). Taken together, the severity of the *O. volvulus* infection as deduced from the microfilarial count or from the nodule does not clearly correlate with the NATOG content in the urine.

### Additional metabolite biomarkers from urine samples of infected and non-infected individuals

Two data sets were compared using the XCMS software to search for metabolite biomarkers for onchocerciasis in the urine samples [20]: (i) non-infected vs. Ov-infected and (ii) all-controls vs. all-Ov-infected. The XCMS analysis of the non-infected vs. Ov-infected samples resulted in the identification of 1299 features (defined by *m*/*z* and retention time, including isotope peaks and different adducts), 89 with a *P* value < 0.01 (Additional file 5: Table S2). In this approach, the *P*-value of NATOG (M356T20) was at position 69 of all features organized by *P*-value (tstat = − 2.78153, *P* = 0.007). Comparison of all-controls vs. all-Ov-infected samples resulted in the identification of 1234 features with 106 compounds with *P* < 0.01. NATOG (M356T20) was at position 42 of all features organized by *P*-value (tstat = 3.4171, *P* = 0.001) (Additional file 6: Table S3). Therefore, these two approaches resulted in the identification of a number of potential biomarkers, including the previously identified NATOG.

**Fig. 2:**
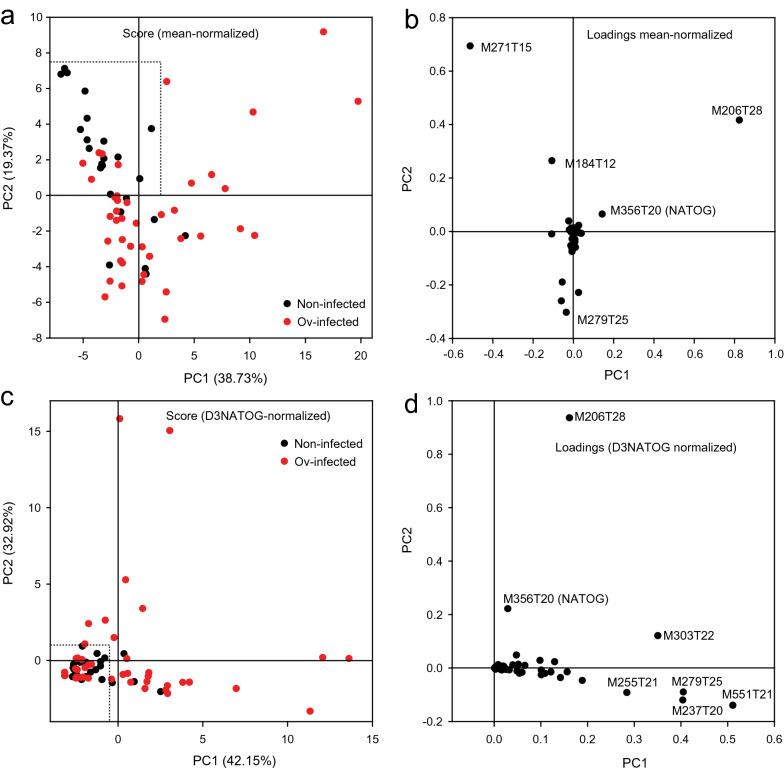
Principal component analysis (PCA) of non-infected and Ov-infected samples. The contents of the features (LC-MS peaks with specific* m*/*z* and retention time) were normalized to the mean of all metabolites (**a**, **b**) or to D_3_-NATOG (**c**, **d**). Features with *P* < 0.01 were used for PCA. **a**, **c** The score plots and **b**, **d** the loadings of the components PC1 and PC2. The dashed boxes in **a** and **c** indicate the thresholds used to score for infected vs. non-infected. The numbers in parentheses in the axes (**a**, **c**) indicate the contribution of the component to total variability in percent

Principal component analysis (PCA) can help to extract information from complex metabolite measurements for the separation of different groups of individuals. For this approach, the peak areas of the features of non-infected vs. Ov-infected samples obtained from the XCMS measurements were normalized to the mean of all features. Next, the data were re-organized according to *P*-values calculated from the normalized data. Peaks outside of the LC gradient time (*t* < 4 min; *t* > 30 min), ^13^C isotope peaks and peaks with *m*/*z* < 100 were eliminated. The features with *P* < 0.01 (~ 50) were used for PCA analysis of the non-infected vs. Ov-infected samples. The score plot of PC1 and PC2 of the data normalized to the mean of all features showed differential clustering of non-infected and Ov-infected samples (Fig. 2a). Using the thresholds as indicated by the dashed box, 18 non-infected and 34 Ov-infected were correctly identified, while 6 non-infected and 7 Ov-infected were not. The features with the highest loadings for PC1 or PC2 were M271T15, M184T12, M356T20 (NATOG) and M206T28 (Fig. 2b). Similarly, the score plot for non-infected vs. Ov-infected data normalized to D_3_-NATOG revealed clustering of 18 non-infected samples using the thresholds indicated by the dashed box, while 29 Ov-infected samples were outside of these thresholds (Fig. 2c). Twelve Ov-infected and six non-infected samples were not correctly identified. Features with high loadings in PC1 or PC2 were M206T28, M551T22, M279T25, M237T20, M303T22, M255T21 and M356T20 (NATOG) (Fig. 2d). Taken together, PCA analysis of data normalized to the mean or to D_3_-NATOG resulted in the separation of non-infected and Ov-infected samples, albeit with some overlap. Two features with considerable loadings for PC1 or PC2 were found in the two approaches, M356T20 (NATOG) and M206T28. In the non-normalized data set, these two features were also elevated in the Ov-infected samples, while they were low in the non-infected controls, and they also showed high loading for the components PC1 and PC2 in PCA analysis of non-normalized samples (not shown), indicating that they might provide discriminative capacity independent of the method of normalization.

### The feature M206T28 corresponds to the urine metabolite cinnamoylglycine

**Fig. 3:**
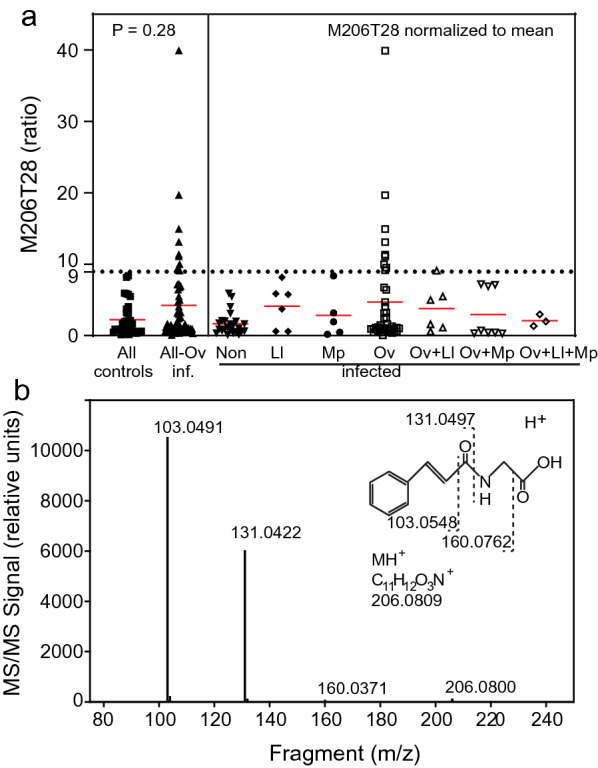
Characterization of the feature M206T28 from urine samples of infected patients. **a** M206T28 normalized to the mean of all features. The dotted line depicts the threshold of 9 (ratio) for *O. volvulus* infections. The red lines show the means of the corresponding data. Significance calculated with Mann-Whitney U test, GraphPad Prism ver 9.1. **b** MS/MS spectrum of the feature M206T28. A sample with high content of M206T28 was analyzed by LC-MS on the Q-TOF instrument with fragmentation of the precursor *m*/*z* 206.0800 at 20 V. The spectrum exactly matches that of cinnamoylglycine as found in the Metlin database

The concentration of the feature M206T28 in urine normalized to the mean of all features is shown in Fig. 3a. When normalized to the mean, the median of the all-Ov-infected samples was not significantly different compared with all-controls (Mann-Whitney U test: *U* = 877, *P* = 0.28). The M206T28 content in Ov-infected (mean ± SEM; 4.7 ± 0.3) was increased compared with non-infected samples (1.7 ± 0.3), The mean content of all-oncho samples (4.2 ± 0.8) was also increased compared with the all-control samples (2.3 ± 0.8). When the threshold was set at the highest value of the all-control samples (9.0), all of the all-control samples were below this threshold, but 10 out of 58 all-Ov-infected samples (17.2%) were higher. Therefore, M206T28 is suitable to identify infected individuals with very high M206T28 levels, but a large variation in the M206T28 contents was observed.

To identify the structure of the feature M206T28, a sample with high M206T28 content was separated by LC-MS on the Q-TOF instrument with fragmentation of the precursor at *m*/*z* 206.0800. The spectrum was used for an MS/MS spectrum match search at the Metlin database [22]. The MS/MS spectrum had characteristic fragments at *m*/*z* 160.0371, 131.0422 and 103.0491. These peaks matched with the spectrum of cinnamoylglycine (CAS 16534-24-0) with a Metlin score of 100 (which is the maximal possible score) (Fig. 3b). Therefore, the feature M206T28 was identified as cinnamoylglycine.

### Analysis of metabolite biomarkers in individuals suffering from multiple infections

**Fig. 4:**
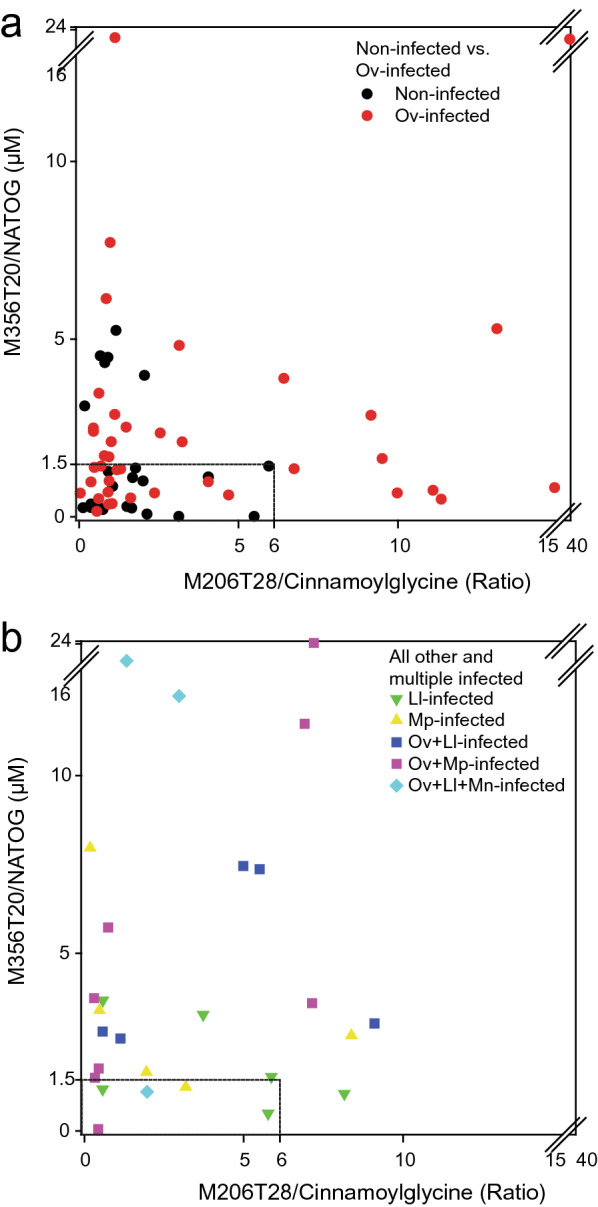
Data separation by two features (LC-MS peaks with specific *m*/*z* and retention time) M356T20 and M206T28. The concentration of M356T20 (NATOG, in µM) and the data normalized to the means of all features of M206T28 were plotted. **a** Results of non-infected vs. Ov-infected. **b** Results of Ll-infected, Mp-infected and multiple-infected samples. The boxes indicate the thresholds of 1.5 µM NATOG and 6 (relative units) of cinnamoylglycine used to score for infected vs. non-infected samples

The results for the features M356T20 (NATOG) and M206T28 (cinnamoylglycine) were plotted for the sets of non-infected and Ov-infected samples (Fig. 4a). When thresholds of 1.5 µM and 6 (ratio) (dashed box in Fig. 4a) were selected for M356T20 and M206T28, respectively, 16 non-infected and 25 Ov-infected samples were identified correctly, while 6 non-infected and 16 Ov-infected samples were not.

To study the prediction capacity of the two features M356T20 (NATOG) and M206T28 (cinnamoylglycine), the levels for the Ll-infected, Mp-infected, Ov + Ll-infected, Ov + Mp-infected and Ov + Ll + Mp-infected samples were compared with the data sets of non-infected and Ov-infected samples. Figure 4b shows that most of the Ll- and Mp-infected samples and almost all of the samples with multiple infections lie outside of the area determined by the thresholds for non-infected individuals (dashed box in Fig. 4b). Only two Ll-infected and one sample each of Mp-infected, Ov + Mp-infected and Ov + Ll + Mp-infected were found below the thresholds. This finding is in agreement with the scenario that the two biomarkers are also elevated in Ll-infected and Mp-infected samples and that they can be used to identify individuals with multiple infections. While most of the other Ll-infected samples had low M356T20 and M206T28 contents, most of the Mp-infected and the multiple-infected samples had highly elevated amounts of the two features.

## Discussion

Onchocerciasis represents a major health risk in Western and Central Africa. Although mass treatment with ivermectin and other measures have considerably contributed to controlling the disease, strategies targeted at the elimination of onchocerciasis are hampered by the lack of reliable diagnostic tools. Different strategies were tested to develop a reliable biomarker for onchocerciasis, focusing on RNA, proteins or metabolites in the blood or urine of infected individuals. The most prominent compound analyzed to date is NATOG, a glucuronide of *N*-acetyltyramine which was detected in the urine of Ov-infected individuals [10]. The mean content of NATOG in onchocerciasis-positive samples from Africa was elevated (36.89 ± 3.99; mean ± SEM, *n* = 81) compared with non-infected controls (6.95 ± 2.74; *n* = 16) [10]. In 2017, Globisch et al. [13] defined a threshold of 13 µM NATOG for onchocerciasis after screening additional *O. volvulus* positive individuals (42.8 ± 3.7 µM, *n* = 145) and non-infected individuals (6.4 ± 0.7 µM). Later, Shirey et al. [14] presented additional NATOG measurements by lateral flow immunoassay, which resulted in NATOG levels in infected individuals (39.2 ± 2.5 µM, *n* = 293) and non-infected individuals (9.29 ± 0.95 µM, *n* = 255) [14]. In these three studies, only limited information on individual patients was presented. Therefore, it remained unclear whether the NATOG levels can be employed to differentiate between infected and non-infected individuals.

In a study by Lagatie et al. [15], NATOG was measured in a different set of urine samples which resulted in lower mean concentrations for infected (1.06 ± 0.16 µM, *n* = 98), non-infected endemic (0.99 ± 0.8 µM, *n* = 51) and non-infected individuals from Europe (0.66 ± 0.18, *n* = 18). These authors came to the conclusion that it is difficult to use NATOG as a diagnostic marker to distinguish infected from non-infected individuals.

Recently, Hotterbeekx et al. [16] conducted a further study on the viability of NATOG as a biomarker for onchocerciasis. The mean amounts of NATOG were elevated in infected (9.7 ± 1.4 µM, *n* = 117) compared with non-infected individuals (3.2 ± 0.9 µM, *n* 55). When the infected patients were grouped according to the severity of epilepsy (‘nodding syndrome’), a known onchocerciasis-associated symptom, it turned out that the NATOG levels increased with increasing severity of epilepsy (3.7 ± 0.9 µM, *n* = 20 infected without epilepsy; 6.1 ± 1.2 µM, *n* = 81, mild forms; 12 ± 3 µM, *n* = 45 severe forms). Furthermore, the group of patients with severe forms of epilepsy had an increased number of nodules. Using the cutoff of 13 µM as previously suggested [13], a low discriminative power of NATOG was recorded on an individual basis, because most NATOG values of infected or non-infected individuals were below 13 µM.

In our study, we recorded a NATOG content (3.02 ± 0.69 µM, *n* = 41; mean ± SEM) in Ov-infected individuals which was increased compared to non-infected samples (1.50 ± 0.35 µM, *n* = 24). The Ll-infected samples (1.87 ± 0.53 µM, *n* = 6) were similar to the non-infected ones, while Mp-infected samples also had elevated NATOG levels (3.38 ± 1.21 µM, *n* = 5), and NATOG in the samples with multiple infections was even higher (Ov + Ll-infected, 11.45 ± 6.86, *n* = 6; Ov + Mp-infected, 6.38 ± 2.71, *n* = 8; Ov + Ll + Mp-infected, 10.00 ± 4.63, *n* = 3). However, when looking on an individual basis, it was difficult to distinguish between infected and non-infected samples. We noticed considerable variability of NATOG (normalized to the internal standard D_3_-NATOG). In addition, the standard D_3_-NATOG itself showed a considerable variation, which was in part due to ion suppression as revealed by injecting diluted samples. However, since the NATOG measurements were always normalized to the D_3_-NATOG, the quantification of NATOG per se was not affected. Attempts to normalize the NATOG contents to other parameters, i.e. creatinine, did not lead to a reduction in variability. In addition, normalization of NATOG levels to the mean or median of all features in the chromatogram did not improve the measurement. Furthermore, we could not detect a correlation of NATOG levels with the number of microfilariae or nodules in the different urine samples, independent of normalization. Therefore, the origin for the high variability of NATOG in non-infected and infected individuals remains unclear.

All of the individuals without *O. volvulus* infection had NATOG levels < 10 µM, a value close to the threshold of 13 µM previously defined by Globisch et al. [10], while 8/58 (13.8%) of the all-Ov-infected individuals had NATOG levels > 10 µM (Fig. 1a). In Table 6 of their manuscript, Hotterbeekx et al. [16] compiled the results of NATOG measurements in urine samples of the previous studies. The maximal amounts of NATOG in samples of all non-infected individuals, of all Mp-infected and Ll + Mp-infected individuals was < 80 µM (the only exception being Ll-infected), while the groups of *O. volvulus*-infected individuals (Ov and co-infection; Ov infection with/without epilepsy) contained data points with NATOG levels > 80 µM [13, 16]. A high level for NATOG could therefore be used to conduct larger epidemiological investigations to determine whether onchocerciasis is still prevalent in a certain area and could even specify a threshold for the elimination on this basis, provided that the results are confirmed, including in areas with lower onchocerciasis endemicity.

PCA analysis revealed that onchocerciasis exerts a complex effect on the metabolite pattern in urine. The score plots of PC1 and PC2 for the metabolite measurements showed a separation into non-infected and Ov-infected samples, with several features including M356T20 (NATOG) and M206T28 revealing high loadings. The use of a combination of NATOG and M206T28 as biomarkers still resulted in high variation for non-infected and Ov-infected samples. However, most samples from Ll-infected, Mp-infected or multiple-infected individuals were correctly assigned (Fig. 4). The structure of the feature M206T28 was identified as cinnamoylglycine based on its MS/MS spectrum (Fig. 3). Cinnamoylglycine is a known metabolite in urine [23]. It is presumably derived by conjugation with glycine from cinnamic acid taken up from a plant-derived diet. The gut microbiota contributes to cinnamoylglycine accumulation, because antibiotic treatment of mice which affects the gut microbiota results in a decrease in cinnamoylglycine in the urine [24], and blood plasma from germ-free mice contained low cinnamoylglycine levels compared with control animals [25]. It is possible that low cinnamoylglycine levels in the urine of some individuals are caused by a previous antibiotic treatment. However, no information on the history of drug or antibiotic administration of the individuals described in this study was available (Additional file 1: Table S1). On the other hand, it has previously been shown that cinnamoylglycine levels in the urine of mice are downregulated after activation of the peroxisome proliferator-activated receptor α (PPARα) with the artificial activator Wy-14,643 [26]. PPARα is a member of the nuclear receptor superfamily involved in the regulation of lipid and glucose metabolism. Furthermore, formaldehyde treatment also resulted in the decrease in cinnamoylglycine in the urine of mice, possibly via PPARα activation [27]. The mechanism of cinnamoylglycine regulation in urine by PPARα or formaldehyde remains unclear. In this aspect, it is interesting to note that PPARα not only regulates metabolic pathways in the liver, heart and muscles, but is also involved in keratinocyte proliferation and differentiation in the skin, wound healing and inflammation [28]. Inflammation of the skin causes a decrease in PPARα expression [28]. Therefore, it is possible that microfilarial infections in the skin and subcutaneous tissue might affect PPARα expression and result in an increased cinnamoylglycine content in the urine.

## Conclusions

In conclusion, onchocerciasis causes complex changes in the urine metabolome with some metabolites, e.g. NATOG and cinnamoylglycine, with elevated amounts in infected individuals. However, the levels of urine metabolites show large variation making it difficult to distinguish infected and non-infected samples. Previous results indicated that severe forms of epilepsy, a known onchocerciasis-associated syndrome, are accompanied by very high levels of NATOG. Another metabolite elevated in infected samples, cinnamoylglycine, can be affected by changes in the gut microbiota due to antibiotic administration. Therefore, additional information on the individuals used for onchocerciasis screening, including data on epilepsy/nodding disease, filarial load and nodule number, as well as drug/antibiotic administration, is highly important to correctly evaluate the metabolite data.

High NATOG and cinnamoylglycine levels proved valuable as a potential cutoff for high specificity (and had at least a sensitivity high enough to inform) during surveys collecting urine samples, without painful skin snip analysis of microfilaridermia, to determine whether onchocerciasis is present in a given area. This might allow developing NATOG and cinnamoylglycine testing as epidemiological markers for geographical mapping of onchocerciasis and during elimination surveys (e.g. TAS). Thus, in mapping endemic areas with O-150 serological testing to begin mass drug treatment or for deciding to end mass treatment, measuring NATOG and/or cinnamoylglycine in matched urine samples could provide a tool to make decisions about borderline results (when the range in CI is so large that it is not possible to determine whether a region/area is below the 2% and 1% cutoffs for mapping and stopping activities, respectively) [29]. Even though most *O. volvulus* infections will not be detected, positivity for high NATOG and/or high cinnamoylglycine has enough discriminative power for programs to conclude whether there is sufficient onchocerciasis prevalence based on the proportion of residents analyzed with O-150 serology. This will allow for faster decision making without the need for a second-stage mapping with larger samples sizes and the associated financial and logistical costs. We will test this hypothesis and establish an initial cutoff, with the aid of modeling, using urine from an upcoming study funded by COR-NTD that will compare O-150 serology to the O-150 LAMP assay [30].

## Supplementary Information

**Additional file 1: Table S1.** Details of urine samples from Cameroon.

**Additional file 2: Text 1.** Synthesis of deuterated *N*-acetyl-tyramine-*O*-β-glucuronide (D3-NATOG).

**Additional file 3: Figure S1.**^1^H-NMR and ^13^C-NMR spectra of *N*-D_3_-acetyltyramine-*O*,β-glucuronide (D_3_-NATOG) and intermediates of synthesis. **a** ^1^H-NMR spectrum of *N*-D_3_-acetyltyramine. **b** ^13^C-NMR spectrum of N-D_3_-acetyltyramine. **c** ^1^H-NMR spectrum of *N*-D_3_-acetyltyramine-*O*-(tri-*O*-acetyl-β-glucuronide methyl ester). **d** ^13^C-NMR spectrum of *N*-D_3_-acetyltyramine-*O*-(tri-*O*-acetyl-β-glucuronide methyl ester). **e** ^1^H-NMR spectrum of *N*-D_3_-acetyltyramine-*O*,β-glucuronide (D3-NATOG). **f** ^13^C-NMR spectrum of *N*-D_3_-acetyltyramine-*O*,β-glucuronide (D3-NATOG). **g** ^1^H-2D-NOESY spectrum of *N*-D_3_-acetyltyramine-*O*,β-glucuronide (D_3_-NATOG). This spectrum proves the exclusive presence of the β-anomer of the glycoside.

**Additional file 4: Figure S2.** Calibration curve for D3-NATOG. Different amounts of D3-NATOG were spiked into a control urine sample and used for D3-NATOG quantification by LC-MS/MS. Means ± SD, *n* = 3.

**Additional file 5: Table S2.** XCMS results for the comparison of Ov-infected vs. non-infected individuals.

**Additional file 6: Table S3.** XCMS results for the comparison of all-Ov-infected vs. all-control individuals.

## Data Availability

All data generated or analyzed during this study are included in this published article and its additional information files.

## Abbreviation

NATOG: *N*-Acetyltyramine-*O*,β-glucuronide
