# Supplementary material for: Urine metabolites for the identification of Onchocerca volvulus infections in patients from Cameroon
Source: Parasit Vectors. 2021 Aug 11;14:397. doi: 10.1186/s13071-021-04893-1 (PMC8359580; doi:10.1186/s13071-021-04893-1)
Supplement: Supplementary file 2 — Additional file 2: Text 1. Synthesis of deuterated N-acetyl-tyramine-O-β-glucuronide (D3-NATOG). [file 13071_2021_4893_MOESM2_ESM.docx]

**Additional file 2: Text 1.** Synthesis of deuterated N-acetyl-tyramine-O-β-glucuronide (D_3_-NATOG).

N-Acetyl-tyramine-O-β-glucuronide harboring three deuterium atoms (D_3_-NATOG) was synthesized in three steps from tyramine and acetobromo-α-D-glucuronic acid methyl ester following an optimized literature procedure (Globisch et al., 2013). D_6_-acetic anhydride (500 µL, 5.28 mmol, 1 eq.) was added dropwise for 10 min to a suspension of tyramine (725 mg, 5.28 mmol, 1 eq.) in solution of dry THF (40 mL) with triethylamine (Et_3_N) (2.2 mL) at 0 C° under argon gas and stirred for 3 h. Next, the solvent was removed *in vacuo*. Purification by flash column chromatography (SiO_2_, ethylacetate + 5% NH_3_ (aq)) afforded a pure compound N-D_3_-acetyltyramine (Additional file 2: Figure S1a, b) (901.00 mg, 4.94 mmol, 94%) as a white powder: **^1^H NMR** (500 MHz, Methanol-d_4_) δ 7.04 (d, J = 8.5 Hz, 2H, H-3, H-5), 6.72 (d, J = 8.5 Hz, 2H, H-2, H-6), 3.35 (t, overlapped with solvent peak, 2H, H-8, H-8’), 2.70 (t, J = 7.4 Hz, 2H, H-7, H-7’); **^13^C-NMR** (125 MHz, CH_3_OD) δ 173.24 (C-10), 156.91 (C-1), 131.22 (C-4), 130.67 (C-5), 116.21 (C-6), 42.38 (C-8), 35.65 (C-7); **MS** (EI): calculated for [M^+·^]: 182.1129, found: 182.1134.

N-D_3_-acetyltyramine (1.20 g, 6.58 mmol, 1.00 eq.) as prepared above and acetobromo-α-D-glucuronic acid methyl ester (3.03 g, 7.24 mmol, 1.10 eq.) were dissolved in dry CH_2_Cl_2_ (185 mL) and stirred for 30 min at room temperature over 4-Å molecular sieves under argon gas. Then, silver trifluoromethanesulfonate (AgOTf) (1.86 g, 7.24 mmol, 1.10 eq.) was added and the reaction mixture was stirred for 3 h. Afterward, CH_2_Cl_2_ was added and filtered through celite, and the solvent was removed *in vacuo*. Purification by flash column chromatography (SiO_2_, CH_2_Cl_2_/MeOH 35:1) afforded N-D_3_-acetyltyramine-O-(tri-O-acetyl-β-glucuronide methyl ester as a colorless oil in a 10:1 ratio of the β/α anomers. Separation of both isomers was performed by preparative HPLC purification (Daicel Chiralpak IB (250x20 mm, 5 µm), n-hexane (HPLC grade)/ ethanol (absolute), 80:20, flow 18.0 mL/min) to obtain pure β-anomer (323.00 mg, 647.94 µmol, 10%), which was used for the deprotection step (Additional file 2: Figure S1c, d): **^1^H-NMR** (700 MHz, chloroform-d) δ 7.12 (d, J = 7.7 Hz, 2H, H-13, H-15), 6.95 (d, J = 7.7 Hz, 2H, H-12, H-16), 5.46 (s, 1H, N-H), 5.36 – 5.32 (m, 2H, H-2, H-3), 5.27 (td, J = 7.2, 2.8 Hz, 1H, H-1), 5.12 (d, J = 7.5 Hz, 1H, H-6), 4.17 (d, J = 7.3 Hz, 1H, H-4), 3.74 (s, 3H, H-10), 3.48 (d, J = 6.0 Hz, 2H, H-18), 2.77 (t, J = 6.7 Hz, 2H, H-17), 2.06 (s, 3H, H-26), 2.05 (s, 3H, H-30), 2.04 (s, 3H, H-34); **^13^C-NMR** (125 MHz, CDCl_3_) δ 170.46 (C-20), 170.10 (C-29), 169.35 (C-25), 169.23 (C-33), 166.89 (C-7), 155.39 (C-11), 133.96 (C-14), 129.99 (C-13), 117.46 (C-12), 99.29 (C-6), 72.87 (C-4), 71.89 (C-2), 71.05 (C-1), 69.16 (C-3), 53.15 (C-10), 40.91 (C-18), 34.86 (C-17), 20.66 (C-26), 20.63 (C-30), 20.53 (C-34); **HRMS** (ESI+), (6.0 eV) m/z calculated for [M+H^+^]: 499.2002, found: 499.1999.

Finally, the protected sugar prepared above (200.00 mg, 401.20 µmol, 1.00 eq.) in H_2_O (50 mL) was treated with 1 M NaOH (10 mL) for 2.5 h. The solution was neutralized with 1 M HCl and dried under reduced pressure. Separation by HPLC (Knauer Eurospher II 100-5 C18 (250x20 mm, 5 µm), acetonitrile + 0.05 vol% trifluoroacetic acid (TFA)/ water (PureLab) + 0.05 vol% TFA, 10:90, flow 24.0 mL/min) and lyophilization of the aqueous solution afforded pure final product D_3_-NATOG (Additional file 2: Figure S1e, f) (80.00 mg, 223.24 µmol) in a yield of 56%: **^1^H-NMR** (700 MHz, methanol-d4) δ 7.15 (d, J = 8.6 Hz, 2H, H-15, H-17), 7.02 (d, J = 8.6 Hz, 2H, H-14, H-18), 4.93 (dd, J = 5.5, 2.2 Hz, 1H, H-4), 3.95 (d, J = 9.8 Hz, 1H, H-6), 3.61 (ddd, J = 9.8, 6.2, 3.0 Hz, 1H, H-1), 3.51 – 3.47 (m, 2H, H-2, H-3), 3.36 (t, J = 7.3 Hz, 2H, H-20, H-20’), 2.74 (t, J = 7.3 Hz, 2H, H-19, H-19’); **^13^C-NMR** (176 MHz, CH_3_OD) δ 173.28 (C-22), 172.14 (C-10), 157.49 (C-13), 134.79 (C-16), 130.73 (C-15, C-17), 118.03 (C-14, C-18), 102.65 (C-4), 77.34 (C-2), 76.57 (C-6), 74.59 (C-3), 73.01 (C-1), 42.15 (C-20), 35.67 (C-19); **HRMS** (ESI+), (6.0 eV) m/z calculated for [M+Na^+^]: 381.1348, found: 381.1341. The anomeric purity was proven by a ^1^H-2D-NOESY spectrum (Additional file 2: Figure S1g).
