# Supplementary material for: Urine metabolites for the identification of Onchocerca volvulus infections in patients from Cameroon
Source: Parasit Vectors. 2021 Aug 11;14:397. doi: 10.1186/s13071-021-04893-1 (PMC8359580; doi:10.1186/s13071-021-04893-1)
Supplement: Supplementary file 3 — Additional file 3: Figure S1.1H-NMR and 13C-NMR spectra of N-D3-acetyltyramine-O,β-glucuronide (D3-NATOG) and intermediates of synthesis. a 1H-NMR spectrum of N-D3-acetyltyramine. b 13C-NMR spectrum of N-D3-acetyltyramine. c 1H-NMR spectrum of N-D3-acetyltyramine-O-(tri-O-acetyl-β-glucuronide methyl ester). d 13C-NMR spectrum of N-D3-acetyltyramine-O-(tri-O-acetyl-β-glucuronide methyl ester). e 1H-NMR spectrum of N-D3-acetyltyramine-O,β-glucuronide (D3-NATOG). f 13C-NMR spectrum of N-D3-acetyltyramine-O,β-glucuronide (D3-NATOG). g 1H-2D-NOESY spectrum of N-D3-acetyltyramine-O,β-glucuronide (D3-NATOG). This spectrum proves the exclusive presence of the β-anomer of the glycoside. [file 13071_2021_4893_MOESM3_ESM.docx]

b

a

c

d

f

e

g

**Additional file 3: Figure S1.** ^1^H-NMR and ^13^C-NMR spectra of N-D_3_-acetyltyramine-O,β-glucuronide (D_3_-NATOG) and intermediates of synthesis. **a** ^1^H-NMR spectrum of N-D_3_-acetyltyramine. **b** ^13^C-NMR spectrum of N-D_3_-acetyltyramine. **c** ^1^H-NMR spectrum of N-D_3_-acetyltyramine-O-(tri-O-acetyl-β-glucuronide methyl ester). **d** ^13^C-NMR spectrum of N-D_3_-acetyltyramine-O-(tri-O-acetyl-β-glucuronide methyl ester). **e** ^1^H-NMR spectrum of N-D_3_-acetyltyramine-O,β-glucuronide (D3-NATOG). **f** ^13^C-NMR spectrum of N-D_3_-acetyltyramine-O,β-glucuronide (D3-NATOG). **g** ^1^H-2D-NOESY spectrum of N-D_3_-acetyltyramine-O,β-glucuronide (D_3_-NATOG). This spectrum proves the exclusive presence of the β-anomer of the glycoside.
